# Meaningfulness and mortality: exploring the sense of coherence in Eastern Finnish men

**DOI:** 10.1177/14034948231220091

**Published:** 2024-01-11

**Authors:** Ilkka Piiroinen, Tomi-Pekka Tuomainen, Tommi Tolmunen, Ari Voutilainen

**Affiliations:** 1 Institute of Public Health and Clinical Nutrition, University of Eastern Finland, Finland; 2 School of Social Services and Health Care, Tampere University of Applied Sciences, Finland; 3 Department of Adolescent Psychiatry, Kuopio University Hospital, Finland

**Keywords:** Cohort study, sense of coherence, mortality, patient-reported outcomes, middle age, men

## Abstract

**Aims:**

The sense of coherence scale has been shown to have an epidemiological relationship with mortality. This study aimed to investigate how the three components of sense of coherence (meaningfulness, comprehensibility and manageability) and the individual items of these components relate to mortality.

**Methods:**

Eastern Finnish men (*n*=2315) aged 42–60 years at baseline in the 1980s completed a 12-item sense of coherence scale and were followed for 25 years, on average, until death or until the end of 2019. Hazard ratios for mortality were calculated using two models: one adjusted for age and the second for an additional 12 mortality risk factors.

**Results:**

Of the three sense of coherence components, only meaningfulness was associated with all-cause mortality, and in the fully adjusted model, those in the weakest tertile had a 1.14 (95% confidence interval 1.01−1.29, *P*=0.042) times higher hazard ratio for mortality than those in the strongest tertile. Of the individual sense of coherence items, only the first question, ‘How often do you have the feeling that you really don’t care about what is going on around you?’, was associated with all-cause mortality, and in the fully adjusted Cox model, the hazard ratio of weak versus strong was 1.18 (95% confidence interval 1.03−1.36, *P*=0.020).

**Conclusions:**

**The sense of coherence component related to meaningfulness, including its first item, ‘Caring about what goes on around you’, plays a significant role in the association with mortality among middle-aged men in Eastern Finland. This item should be considered a noteworthy patient-reported variable when predicting mortality in public health settings.**

## Introduction

The sense of coherence (SOC) scale is a life-orientation questionnaire with three components: (a) comprehensibility, which is a cognitive component that measures a person’s ability to understand events around them; (b) manageability, which is an instrumental or behavioural component that assesses the extent to which one can manage these events; and (c) meaningfulness, which is a motivational component related to the ability to find meaning in situations [1, 2]. SOC reflects an individual’s capacity to respond to stress in life, and various forms of the SOC scale have been widely utilised in health-related studies worldwide [3].

Systematic reviews have consistently demonstrated that a strong SOC is linked to better mental health and a better quality of life, acting as a protective factor against depression, burnout, hopelessness and anxiety [3]. Conversely, a weak SOC has been found to increase the risk of all-cause mortality in the general adult population [4]. Furthermore, a decline in SOC during long-term follow-ups has been associated with a higher hazard for all-cause mortality among middle-aged men [5]. However, there is limited knowledge regarding the specific relationships between the three SOC components – comprehensibility, manageability and meaningfulness – and mortality.

Aaron Antonovsky, the developer of the SOC construct, posited that the motivational component of SOC, meaningfulness, holds a central role. He suggested that high motivation could compensate for weak comprehensibility or manageability, whereas strong comprehensibility or manageability might only be temporary without motivation [1]. Antonovsky also emphasised the holistic nature of SOC, in which successful coping depends on all three components [1].

In a large study involving Finnish adults, it was found that due to the superiority of a one-factor model in their sample, the three components should be merged when using a 12-item SOC scale [6]. However, evidence suggests that the comprehensibility component may take precedence as the most critical factor under certain circumstances. For instance, a study of middle-aged participants from Sweden revealed that only the comprehensibility component of a 29-item SOC scale was associated with mortality, with higher scores in comprehensibility correlating with lower mortality rates [7]. In addition, a subsequent study on individuals diagnosed with a first-time acute myocardial infarction using a 13-item scale showed that only the comprehensibility component accounted for observed changes in other SOC components over 2 years [8]. These findings suggest that strong comprehensibility can enhance individuals’ understanding and navigation of challenging situations, thereby reinforcing their overall sense of manageability and meaningfulness [9].

While the relationship between different components of the SOC scale and mortality remains relatively unexplored, even less is known about the association between individual SOC items and health outcomes. While multi-item scales typically result in improved psychometric properties, the inclusion of multiple questions or questionnaires can lead to increased missing data due to the respondent burden [10]. Therefore, understanding which items are most sensitive to a specific outcome becomes valuable [11].

This study aims to assess the associations between the three theory-based components (meaningfulness, comprehensibility and manageability) using the Finnish version of a 12-item SOC scale, along with its items, and all-cause mortality in a cohort of middle-aged Finnish men.

## Methods

### The Kuopio Ischaemic Heart Disease Risk Factor Study

The Kuopio Ischaemic Heart Disease Risk Factor (KIHD) Study is a population-based follow-up study. Originally, in the 1980s, it aimed to investigate the reasons for the high prevalence of coronary heart disease among Eastern Finnish men living in the Northern Savonia District [12]. Later, the KIHD Study broadened its aims to study all aspects of health. The baseline study sample comprised a randomly selected, population-based and age-stratified sample of 2682 men from Eastern Finland, recruited between 1984 and 1989 in two subcohorts. The ages of the participants varied from 42 to 61 years, and the mean age was 53 years. This study included 2315 participants who answered, at least partially, the SOC questionnaire. The research ethics committee of the University of Kuopio gave ethical approval for the KIHD Study on 1 December 1983. All participants gave their informed consent.

### SOC measurements

The KIHD Study utilised a 13-item SOC scale, which demonstrated good structural validity and temporal stability in a large Finnish sample [13]. However, the KIHD Study’s final version of the SOC scale excluded the 10th question from the manageability component (‘Many people, even those with a strong character, sometimes feel like sad sacks in certain situations. How often have you felt this way in the past?’) due to difficulties in translating it from English to Finnish [14]. Consequently, the SOC scale consisted of 12 items (Table I).

**Table I.** Details of the sense of coherence (SOC) scale used in the Kuopio Ischaemic Heart Disease Risk Factor Study.

Component	Item
Meaningfulness	1) How often do you have the feeling that you really don’t care about what is going on around you? (1 = Very seldom or never to 7 = Very often)
Comprehensibility	2) Has it happened in the past that you were surprised by the behaviour of people whom you thought you knew well? (1 = Never happened to 7 = Always happened)
Manageability	3) Has it happened that people whom you counted on disappointed you? (1 = Never happened to 7 = Always happened)
Meaningfulness	4) Until now your life has had: (1 = No clear goals and purpose to 7 = Very clear goals and purpose)
Manageability	5) Do you have the feeling that you are being treated unfairly? (1 = Very often to 7 = Very seldom or never)
Comprehensibility	6) Do you have the feeling that you are in an unfamiliar situation and don’t know what to do? (1 = Very often to 7 = Very seldom or never)
Meaningfulness	7) Doing the things you do every day is: (1 = A source of deep pleasure and satisfaction to 7 = A source of pain and boredom)
Comprehensibility	8) Do you have very mixed-up feelings and ideas? (1 = Very often to 7 = Very seldom or never)
Comprehensibility	9) Does it happen that you experience feelings that you would rather not feel? (1 = Very often to 7 = Very seldom or never)
Comprehensibility	10) When something happened, have you generally found that: (1 = You overestimated or underestimated its importance to 7 = You saw things in the right proportion)
Meaningfulness	11) How often do you have the feeling that there is little meaning in the things you do in your daily life? (1 = Very often to 7 = Very seldom or never)
Manageability	12) How often do you have feelings that you are not sure you can keep under control? (1 = Very often to 7 = Very seldom or never)

Each sense of coherence (SOC) item was rated on a seven-point semantic scale resulting in a total scale score range of 12 to 84. The scores of items 1), 2), 3), and 7) were reversed so that higher scores indicated stronger SOC.

The meaningfulness component of the SOC scale included four items and had an internal reliability of 0.59 measured as Cronbach’s alpha. The comprehensibility component had five items, and Cronbach’s alpha was 0.72. The manageability component contained three items, and Cronbach’s alpha was 0.53.

The respondents rated each SOC item on a seven-point semantic scale, resulting in a total scale score ranging from 12 to 84. For statistical analyses, the negatively worded item scores were reversed, so that for every item, higher scores signified a stronger SOC. The internal reliability of the SOC-12 scale, measured as Cronbach’s alpha, was 0.82.

### Covariates and outcomes

We included covariates besides SOC, such as age and leading somatic risk factors for mortality [15]. We also considered covariates applied in other cohort studies exploring the relationship between SOC and mortality [4]. We did not use psychometrical measurements other than SOC to avoid over-adjustment [16]. Consequently, our final set of covariates included age and the following 12 variables: smoking status, alcohol consumption, years of education, marital status, employment status, history of cancer, history of cardiovascular disease (CVD), physical activity, diabetes, systolic blood pressure (SBP), body mass index (BMI) and serum total cholesterol (STC) [5]. The first seven of these 12 variables were based on the participant’s self-reports and the other five on direct measurements or interviews. Details of these measurements in the KIHD Study are described elsewhere [5, 17].

The outcome variable was all-cause mortality. The follow-up of mortality started at baseline and lasted to death or until 31 December 2019, whichever came first. The average follow-up time was 24.8 years. The Statistics Finland database provided the exact outcome dates of those who died during the follow-up period (data permission number TK-53-1770-16).

### Statistical methods

Using vital status at the end of 2019 as a grouping variable, we analysed the differences between baseline variables with the Student’s *t*-test for the normally distributed continuous variables, with the Mann–Whitney U test for the non-normally distributed continuous variables and with the chi-squared test for the categorical variables. The correlations between the individual SOC items were reported as Spearman’s rank coefficients. We used the following cut-off points to interpret the correlation coefficients: negligible correlation = 0.00–0.10, weak correlation = 0.11–0.39, moderate correlation = 0.40–0.69, strong correlation = 0.70–0.89 and very strong correlation = 0.90–1.00 [18].

The hazard ratios (HRs) of all-cause mortality associated with the sum of SOC items (SOC-12 scale), SOC scale components and individual SOC items were computed with the Cox proportional hazards model. All these measures were recoded into three ordinal groups in the following manner: The SOC scale and its components were recoded as weak = the weakest tertile, medium = middle tertile and strong = the strongest tertile. Individual SOC items were recoded as weak = 1–3 points, medium = 4–5 points and strong = 6–7 points. Strong groups were used as the reference category. Regarding SOC, a similar three-group division has been used in earlier studies [4]. The HR calculations were executed in two steps: model 1 was adjusted for age, and model 2 was adjusted for age and the 12 covariates that were previously determined [5]. Of these covariates, six were continuous (alcohol consumption, physical activity, years of education, SBP, BMI and STC), and six were binomial (smoking status, marital status, employment status, history of cancer, history of CVD and diabetes). To detect time-varying covariates in the Cox model, we tested the proportional hazards assumption [19]. Of the covariates included in the Cox models, age, smoking status and a history of CVD violated the proportional hazards assumption – that is, β(t) ≠ c – and they were treated as time-dependent variables.

We used an item-specific mean imputation to replace missing values. Mean imputation is generally the preferred method for imputing missing baseline variables [20]. Those who answered the SOC questionnaire at baseline (*n*=2315), and were thus included in the study, skipped item number 10 most often (*n*=29, 1.3%) and item number 5 least often (*n*=8, 0.4%). On average, there were 16 (0.7%) missing answers per SOC item. Out of the 12 covariates, 58 values (2.5%) were missing. These missing values were imputed using variable-specific means or modes, depending on the scale of the variable. There were no missing data for the outcome variable all-cause mortality. The statistical significance level was set at 0.05. Statistical analyses were conducted using the IBM SPSS Statistics version 27. We used the strengthening the reporting of observational studies in epidemiology (STROBE) cohort checklist when writing our report [21].

## Results

### Cohort characteristics

Table II presents the participants’ baseline characteristics by vital status at the end of 2019. The respective mean (standard deviation) and maximum follow-up times were 24.8 (9.1) and 35.8 years, respectively. All covariates, except for a history of cancer, showed statistically significant differences at baseline between those who were alive (*n*=801, 34.6%) and those who were deceased (*n*=1514, 65.4%).

**Table II.** Characteristics of the Kuopio Ischaemic Heart Disease Risk Factor Study participants (*n*=2315) by vital status at the end of the year 2019.

Variable	Alive	Deceased	*P* value
*n* (%)	801 (34.6)	1514 (65.4)	**<0.001** ^a^
Age years, mean (SD)	50.5 (5.6)	54.5 (4.2)	**<0.001** ^a^
SOC, mean (SD)	63.0 (9.4)	61.9 (9.9)	**0.014** ^a^
Smoker, *n* (%)	153 (19.1)	561 (37.1)	**<0.001** ^b^
Alcohol g/week, median (IQR)	27.0 (66.0)	32.1 (95.0)	**0.015** ^c^
CLPA kcal/day, median (IQR)	95.3 (159.9)	81.1 (161.0)	**0.016** ^c^
Education years, mean (SD)	9.6 (3.7)	8.2 (3.2)	**<0.001** ^a^
Not married, *n* (%)	70 (8.7)	235 (15.5)	**<0.001** ^b^
Retired or no work, *n* (%)	126 (15.7)	585 (38.6)	**<0.001** ^b^
History of cancer, *n* (%)	10 (1.2)	34 (2.2)	0.095^b^
History of CVD, *n* (%)	204 (25.5)	667 (44.1)	**<0.001** ^b^
Diabetes, *n* (%)	15 (1.9)	116 (7.7)	**<0.001** ^b^
SBP mmHg, mean (SD)	130.1 (14.0)	136.0 (18.0)	**<0.001** ^a^
BMI kg/m^2^, mean (SD)	26.3 (3.1)	27.1 (3.7)	**<0.001** ^a^
STC mmol/l, mean (SD)	5.7 (1.0)	6.0 (1.1)	**<0.001** ^a^

SD: standard deviation; SOC: sense of coherence; IQR: interquartile range; CLPA: conditioning leisure-time physical activity; CVD: cardiovascular disease; SBP: systolic blood pressure; BMI: body mass index; STC: serum total cholesterol.

a Student’s *t*-test; ^b^Chi-squared test; ^c^Mann–Whitney U test.

Statistically significant *P* values are set in bold type.

Table III shows the correlations between all SOC-12 items. At most, the correlations reached a moderate level. The strongest correlation (0.67) was between SOC items eight (‘Do you have very mixed-up feelings and ideas?’) and nine (‘Does it happen that you experience feelings that you would rather not feel?’), both of which belonged to the comprehensibility component. The lowest and most negligible correlation (0.09) was between the third (‘Has it happened that people whom you counted on disappointed you?’) and fourth (‘Until now your life has had: 1 = No clear goals and purpose to 7 = Very clear goals and purpose’) SOC items, which belonged to the manageability and meaningfulness components, respectively.

**Table III.** Correlation matrix between the SOC items in the Kuopio Ischaemic Heart Disease Risk Factor Study sample (*n*=2315) measured with Spearman’s rank coefficient.

Item	2.	3.	4.	5.	6.	7.	8.	9.	10.	11.	12.
1.	0.192**	0.140**	0.215**	0.206**	0.233**	0.196**	0.243**	0.216**	0.092**	0.242**	0.218**
2.	–	0.507**	0.128**	0.254**	0.225**	0.139**	0.235**	0.235**	0.185**	0.183**	0.174**
3.	0.507**	–	0.085**	0.325**	0.180**	0.123**	0.205**	0.219**	0.171**	0.157**	0.141**
4.	0.128**	0.085**	–	0.345**	0.373**	0.378**	0.417**	0.390**	0.239**	0.474**	0.418**
5.	0.254**	0.325**	0.345**	–-	0.410**	0.256**	0.411**	0.393**	0.215**	0.401**	0.400**
6.	0.225**	0.180**	0.373**	0.410**	–	0.267**	0.529**	0.520**	0.326**	0.440**	0.514**
7.	0.139**	0.123**	0.378**	0.256**	0.267**	–	0.295**	0.290**	0.143**	0.425**	0.335**
8.	0.235**	0.205**	0.417**	0.411**	0.529**	0.295**	–	0.666**	0.337**	0.497**	0.569**
9.	0.235**	0.219**	0.390**	0.393**	0.520**	0.290**	0.666**	–	0.348**	0.478**	0.528**
10.	0.185**	0.171**	0.239**	0.215**	0.326**	0.143**	0.337**	0.348**	–	0.310**	0.343**
11.	0.183**	0.157**	0.474**	0.401**	0.440**	0.425**	0.497**	0.478**	0.310**	–	0.538**
12.	0.174**	0.141**	0.418**	0.400**	0.514**	0.335**	0.569**	0.528**	0.343**	0.538**	–

The ordinal numbers refer to the sense of coherence (SOC)-12 scale items.

** Correlation is significant at 0.01 level (2-tailed).

### Prospective associations between SOC and all-cause mortality

Table IV presents the HRs for all-cause mortality for the three SOC scale components. Meaningfulness was the only statistically significant component in both models. Individuals in the weakest meaningfulness tertile had a 27% higher age-adjusted HR and a 14% higher fully adjusted HR for mortality compared with those in the strongest tertile.

**Table IV.** Hazard ratios for all-cause mortality with respect to the SOC scale meaningfulness, comprehensibility, and manageability tertiles in the Kuopio Ischaemic Heart Disease Risk Factor Study (*n*=2315).

Component	*n*	HR^1^ (95% CI)	*P* value	HR^2^ (95% CI)	*P* value
Meaningfulness					
Weak	713	1.27 (1.12–1.43)	**<0.001**	1.14 (1.01–1.29)	**0.042**
Medium	750	1.00 (0.88–1.13)	0.994	1.00 (0.88–1.13)	0.982
Comprehensibility					
Weak	717	1.12 (0.99–1.27)	0.077	1.00 (0.88–1.13)	0.998
Medium	780	1.00 (0.89–1.13)	0.951	0.97 (0.86–1.09)	0.583
Manageability					
Weak	667	1.20 (1.06–1.36)	**0.005**	1.02 (0.90–1.16)	0.737
Medium	800	1.06 (0.94–1.20)	0.332	1.03 (0.91–1.16)	0.637

Reference is the strong tertile.

CI: confidence interval; HR: hazard ratio; SOC: sense of coherence.

1 Model 1 adjusted for age.

2 Model 2 adjusted for age, smoking, alcohol consumption, leisure-time physical activity, education years, not married, retired or no work, history of cancer, history of cardiovascular disease, diabetes, systolic blood pressure, body mass index, and total cholesterol concentration.

Statistically significant *P* values are set in bold type.

In the age-adjusted Cox regression model, seven out of the 12 SOC items showed statistically significant associations with all-cause mortality, with HRs ranging from 1.19 to 1.42 (Table V). In the total SOC-12 scale, participants in the weakest tertile had a 26% higher HR for all-cause mortality compared with those in the strongest tertile.

**Table V.** Hazard ratios for all-cause mortality with respect to the SOC scale items, and the complete SOC scale in the Kuopio Ischaemic Heart Disease Risk Factor Study (*n*=2315).

Item	Level	HR^1^ (95% CI)	*P* value	Level	HR^2^ (95% CI)	*P* value
1. SOC	Weak	1.21 (1.05–1.39)	**0.009**	Weak	1.18 (1.03–1.36)	**0.020**
	Medium	1.19 (1.06–1.33)	**0.004**	Medium	1.15 (1.02–1.29)	**0.020**
2. SOC	Weak	0.99 (0.86–1.13)	0.846	Weak	0.94 (0.82–1.08)	0.404
	Medium	1.07 (0.96–1.20)	0.226	Medium	1.02 (0.91–1.15)	0.709
3. SOC	Weak	1.12 (0.98–1.27)	0.101	Weak	1.01 (0.88–1.15)	0.921
	Medium	1.13 (1.00–1.28)	**0.044**	Medium	1.10 (0.98–1.25)	0.116
4. SOC	Weak	1.42 (1.18–1.71)	**<0.001**	Weak	1.12 (0.93–1.36)	0.233
	Medium	1.11 (0.99–1.24)	0.067	Medium	1.01 (0.90–1.13)	0.887
5. SOC	Weak	1.15 (1.01–1.31)	**0.037**	Weak	1.00 (0.88–1.15)	0.960
	Medium	1.09 (0.97–1.22)	0.172	Medium	1.06 (0.94–1.19)	0.368
6. SOC	Weak	1.13 (0.98–1.31)	0.098	Weak	1.03 (0.88–1.19)	0.739
	Medium	1.08 (0.96–1.21)	0.197	Medium	1.02 (0.91–1.14)	0.797
7. SOC	Weak	1.33 (1.07–1.66)	**0.010**	Weak	1.13 (0.90–1.41)	0.302
	Medium	0.99 (0.88–1.10)	0.804	Medium	1.02 (0.91–1.14)	0.716
8. SOC	Weak	1.15 (0.97–1.36)	0.110	Weak	1.02 (0.86–1.20)	0.863
	Medium	1.06 (0.94–1.19)	0.356	Medium	0.97 (0.87–1.09)	0.657
9. SOC	Weak	1.05 (0.89–1.24)	0.543	Weak	0.94 (0.80–1.11)	0.462
	Medium	1.01 (0.91–1.13)	0.809	Medium	0.97 (0.86–1.08)	0.555
10. SOC	Weak	1.06 (0.93–1.21)	0.388	Weak	1.03 (0.90–1.17)	0.715
	Medium	1.07 (0.95–1.21)	0.252	Medium	1.04 (0.92–1.18)	0.504
11. SOC	Weak	1.21 (1.03–1.42)	**0.018**	Weak	1.09 (0.93–1.28)	0.302
	Medium	1.09 (0.96–1.22)	0.175	Medium	1.05 (0.93–1.19)	0.410
12. SOC	Weak	1.28 (0.10–1.50)	**0.002**	Weak	1.15 (0.98–1.34)	0.086
	Medium	1.08 (0.96–1.22)	0.217	Medium	1.02 (0.90–1.15)	0.813
All SOC	Weak	1.26 (1.11–1.43)	**<0.001**	Weak	1.09 (0.96–1.24)	0.199
	Medium	1.00 (0.89–1.13)	0.969	Medium	0.97 (0.86–1.10)	0.649

The reference category is the strong tertile.

CI: confidence interval; HR: hazard ratio; SOC: sense of coherence.

Each SOC item was measured on a seven-point semantic scale: Weak = 1–3 points; Medium = 4–5 points; Strong = 6–7 points.

All SOC = sum of SOC items 1 to 12: Weak = the weakest tertile (*n*=716); Medium = the middle tertile (*n*=866); Strong = the strongest tertile (*n*=733).

1 Model 1 adjusted for age.

2 Model 2 adjusted for age, smoking, alcohol consumption, leisure-time physical activity, education years, not married, retired or no work, history of cancer, history of cardiovascular disease, diabetes, systolic blood pressure, body mass index, and total cholesterol concentration.

Statistically significant *P* values are set in bold type.

In the fully adjusted model, only the first item of the SOC scale (‘How often do you have the feeling that you really don’t care about what is going on around you?’) was found to be associated with all-cause mortality. In particular, compared with participants who answered 6 to 7, those who answered 4 to 5 had a 15% higher HR, and those who answered 1 to 3 had a 18% higher HR for all-cause mortality. When the first SOC item was used as a continuous covariate in the Cox model, the per-point HR for all-cause mortality was 1.05 (95% confidence interval (CI) 1.01−1.08, *P*=0.009).

## Discussion

This study examined the association between a Finnish version of the 12-item SOC scale, its three components and individual items and all-cause mortality in a cohort of middle-aged Finnish men. Our findings align with the SOC theory of Antonovsky, highlighting the meaningfulness component as the most significant predictor of all-cause mortality [1]. In addition, we made a novel discovery. The first question on the SOC-12 scale, which assesses how much individuals care about what goes on around them, emerged as the most prominent factor in relation to mortality. Notably, this question is also part of the meaningfulness scale.

The total scores for the SOC-12 scale did not show an association with mortality in the fully adjusted model. This finding contradicts the pooled results of a previous meta-analysis [4], suggesting potential methodological differences between the studies. However, another Finnish study [22] used an almost identical SOC scale to ours and was otherwise highly comparable to our study, demonstrating an association between an overall SOC and all-cause mortality among men, even in a model adjusted for covariates similar to ours. The differences between our study and theirs included a larger number of male participants (2315 vs. 3850), a wider age range (42–60 vs. 25–74 years) and a different geographical sample presentation (Eastern Finland vs. the entire country) [22]. A larger or more diverse sample might have revealed statistically significant differences in our study’s fully adjusted model HRs for mortality. This speculation arises because those who were alive at the end of 2019 had a stronger SOC than those who were not, as indicated in Table II.

Of the three components of the SOC scale, only meaningfulness was associated with mortality in our study. This contradicts earlier findings from Sweden, in which weaker comprehensibility and stronger manageability were associated with higher mortality rates among men [7]. The Swedish study used a 29-item SOC scale. It included older participants, had a smaller sample of male participants (539 vs. 2315), followed the cohort for a shorter time (14 years vs. over 20 years) and used a different set of covariates [7] than our study, all of which could explain the difference. Nevertheless, both of these studies suggest that it may be beneficial to analyse the individual components of SOC separately, contrary to some studies’ suggestions [6].

Of the individual items, only one item in the SOC-12 scale maintained its significance regarding mortality in the fully adjusted model. The reasons for this remain speculative and warrant further investigation for each item. For instance, in the age-adjusted model, the fourth item, which pertains to life goals and purpose, exhibited a clear association with mortality (HR = 1.42, 95% CI 1.18−1.71, *P*=<0.001). However, after additional adjustments, this association diminished (HR = 1.12, 95% CI 0.93−1.36, *P*=0.233). Prior research has demonstrated a connection between life purpose and mortality, even in the fully adjusted models, but these studies often had shorter follow-up periods and reported stronger associations among women [23], which differs from our study involving men.

Our study suggests that the first question in the SOC-12 scale, which is also part of the meaningfulness component, exhibits the strongest association with mortality. The item is about caring about what happens in one’s environment. The importance of caring was also highlighted by Antonovsky, who stated, ‘Without caring one soon comes to fall behind in one’s understanding and lose one’s command of resources’ [1]. He reasoned that with strong meaningfulness, one would be motivated to seek resources and thus improve both comprehensibility and manageability [1]. An earlier study conducted among first-time acute myocardial infarction patients challenged the significance of meaningfulness, as only comprehensibility predicted development in the other two components during a 2-year follow-up [8]. This suggests that shortly after a traumatic experience, comprehension may be the most crucial factor. We did not follow the development of the SOC components, but our study had a much longer follow-up period spanning over 20 years and included a more heterogeneous sample that was not defined by a traumatic event. Our findings suggest that among a random sample of middle-aged Finnish men, caring within the meaningfulness component may play a significant role in driving them and supporting their long-term health. Moreover, there is a suggestion that psychiatric symptoms could either mediate or moderate the connection between SOC and mortality [24]. This idea can also be extended, especially when considering the relationship between meaningfulness and ‘Caring about what goes on around you’, and its association with mortality.

Based on our findings, the significance of caring appears to stand out when compared with the other three items of meaningfulness in the SOC-12 scale, including life goals, daily satisfaction and a sense of meaning in daily life. Our results imply that, from a health perspective, individuals may not necessarily need well-defined life goals, daily enjoyment or a pervasive sense of meaning in everything they do if they possess a strong sense of caring. Our findings indicate that the first SOC item should be included in patient-reported outcome-item libraries [10] and banks [11].

As the primary strength of this study, we utilised data from a regionally representative cohort study of middle-aged men [4], allowing us to incorporate several important covariates [4, 5] into the survival analysis. In addition, our study benefitted from an exceptionally long follow-up period of over 20 years, enabling us to observe both short-term fluctuations in mortality and long-term development.

One important limitation of our study is the limited generalisability of our results, as they were based solely on a baseline cohort of middle-aged men from Eastern Finland during a specific cultural period. Even among the provinces in Finland, there are variations in morbidity. Eastern Finland, especially Northern Savo, maintains the highest morbidity index in Finland [25], which aligns with the early findings of the KIHD Study in the 1980s [14]. Therefore, future studies should seek to validate our results in diverse cohorts and across various countries.

## Conclusions

In summary, our study underscores the central role of the meaningfulness component within the SOC scale in its association with all-cause mortality among middle-aged men in Eastern Finland. Notably, we identified the first item in the meaningfulness component as particularly significant, indicating that a lack of care about what goes on around us can be associated with a heightened mortality hazard. Based on these findings, it may be worth considering the integration of the SOC meaningfulness component, with particular attention being paid to its initial item, into patient-reported measures aimed at predicting mortality. However, further research is warranted to validate and reinforce these conclusions.
